# Effect of Electroacupuncture on Reproductive Disorders and Insulin Resistance in a Murine Polycystic Ovary Syndrome Model

**DOI:** 10.1155/2021/9968463

**Published:** 2021-12-26

**Authors:** Chang Ji, Wanling Xu, Zhiqing Zhang, Shuai Cui, Wei Yi

**Affiliations:** ^1^South China Research Center for Acupuncture and Moxibustion, Medical College of Acu-Moxi and Rehabilitation, University of Chinese Medicine, Guangzhou 510006, China; ^2^Acupuncture Research Team, The Second Affiliated Hospital of Guangzhou University of Chinese Medicine, Guangzhou, China; ^3^Research Institute of Acupuncture and Meridian, Anhui University of Chinese Medicine, Hefei, China

## Abstract

Polycystic ovarian syndrome (PCOS) is a common, complex, and heterogeneous endocrine and metabolic disorder. There is no standardized treatment, and it therefore requires individualized therapies according to the symptoms and pathogenesis of each patient. The present study aimed to determine the effect of electroacupuncture at the acupoints Zusanli (ST36), Sanyinjiao (SP6), and Neiguan (PC6) on reproductive disorders and insulin resistance in a murine model of PCOS induced by dehydroepiandrosterone (DHEA). Vaginal smear analysis was used to determine mice estrous cycle; intraperitoneal glucose and insulin tolerance tests were adopted to analyze metabolic characteristics; enzyme-linked immunosorbent assay was used to measure hormone levels; gene expression was quantified with real-time PCR; hematoxylin and eosin staining was used to observe ovarian morphology. We observed disordered estrous cycle, polycystic ovarian morphology, and higher levels of homeostasis model assessment-insulin resistance (HOMA-IR) and testosterone (T), indicating successful modeling of PCOS. DHEA increased levels of estrogen (E_2_), progesterone (P), testosterone (T), luteinizing hormone (LH), and follicle-stimulating hormone (FSH), and EA treatment restored them to levels seen in the control group. EA reduced the days in estrus caused by DHEA, improved the abnormal sex hormone receptor genes, and attenuated the DHEA-induced histomorphological changes in mouse ovaries. The average expressions of the androgen receptor (AR), estrogen receptor (ER), luteinizing hormone receptor (LHR), and follicle-stimulating hormone receptor (FSHR) genes in the ovary greatly increased after DHEA treatment and significantly decreased in the DHEA + EA group. After EA treatment, the cystic follicle (CF) number was reduced and corpora lutea (CL) increased in the DHEA + EA group compared to the DHEA group. EA improved glucose intolerance and insulin intolerance. Statistical analysis of intraperitoneal glucose tolerance test-area under curve (IPGTT-AUC) glucose levels revealed a significant decrease in DHEA group mice compared to the control and DHEA + EA groups. EA was found to restore fasting blood glucose, fasting serum insulin, and HOMA-IR. In summary, our study suggests that EA has a remarkable effect in the DHEA-induced murine PCOS model. Management of EA could improve estrous cycle, hormonal disorders, abnormal sex hormone receptors in ovaries, ovary morphology, and insulin resistance in PCOS mice.

## 1. Introduction

Polycystic ovarian syndrome (PCOS) is a common but complex and heterogeneous endocrine and metabolic disorder characterized by anovulation or oligoovulation, hyperandrogenism, and polycystic ovarian morphology [1–4]. Approximately 5–10% of reproductive-age women develop PCOS, as a result of either genetics or lifestyle conditions such as being in a dark environment for long periods of time, overeating, or not getting enough exercise [5]. Primary clinical manifestations of PCOS include ovulatory dysfunction, infertility, hirsutism, acne, and obesity [6]. Women with PCOS have more frequent pulses of gonadotropin-releasing hormone (GnRH) and luteinizing hormone (LH), as well as an increased LH/follicle-stimulating hormone (FSH) ratio. At the hypothalamic-pituitary level, progesterone is not able to regulate the secretion of GnRH as it does in women without PCOS [7]. The ovarian theca cells secrete higher levels of androgen because of the increased LH.

According to histological analyses, the ovaries of PCOS patients have numerous antral follicles; however, the etiology needs further research. Reactive oxygen species (ROS) induced by PCOS could cause ovarian damage, and some reports have shown that antioxidants have a positive effect on ovaries [8, 9]. Reports show that PCOS is closely associated with hypothalamus-pituitary-ovarian axis dysfunction, hyperandrogenism, insulin resistance (IR), hyperinsulinemia, and type II diabetes [10, 11]. Although IR is a common pathologic manifestation of PCOS (seen in ∼85% of PCOS patients, especially those who are overweight), it is not one of the diagnostic criteria for PCOS. Research has shown that IR and obesity have a close linkage with PCOS; clinical manifestations of PCOS are more apparent in patients with IR and obesity [12–14]. The relationship between PCOS and IR is still unknown, and further research is required. There are currently no effective cures for PCOS. The development of new therapies with increased effectiveness and fewer side effects remains an urgent need.

There have been reports that both pharmacological and nonpharmacological therapies for PCOS may cause side effects such as cardiovascular disease and multifetation [15, 16]. A great number of clinical trials and animal studies have reported that electroacupuncture (EA) treatment could effectively relieve PCOS symptoms in humans. Jedel et al. found that EA had a better effect in the treatment of hyperandrogenism and oligo/amenorrhea [17] compared to physical exercise. Yu et al. indicated that EA could improve obesity-related indexes and insulin sensitivity of obese PCOS patients [18]. Through a randomized controlled trial, Cao et al. concluded that Tung's acupuncture could regulate menstruation and control weight [19]. Compared with the attention and time involved in meeting with a therapist, 10–13 weeks of acupuncture treatment showed more improvement of ovulation frequency in lean/overweight women with PCOS, suggesting that acupuncture is more effective than placebo [20]. These data illustrate that EA treatment can be used to treat oligo/amenorrhea, obesity, and IR in women with PCOS. However, some randomized clinical trials indicate that acupuncture has no apparent effect on PCOS [21]. In animal experiments, a previous study showed that the pathological symptoms of PCOS in rats, such as serum hormone levels and insulin resistance, were improved after EA treatment [22]. Cui et al. indicated that PCOS rats restored estrous cyclicity, increased numbers of preovulatory follicles and corpora lutea, and decreased body weight after four weeks of EA treatment [23]. In total, the effects of acupuncture in humans and animals with PCOS are controversial, and more experiments are needed to provide clarification.

Previous research has demonstrated that DHEA-induced PCOS models have various representative characteristics such as estrous cycle disorder, impaired sensitivity to glucose and insulin, disruption of hormones, and change in ovarian morphology [24–27]. In the present study, we established a PCOS mouse model by DHEA injection and studied the effects of EA on the model.

## 2. Materials and Methods

### 2.1. Animal Model and Experimental Design

Thirty 21-day-old female C57BL/6J mice were purchased from the Experimental Animal Center of Guangzhou University of Chinese Medicine (SCXK (Yue) 2018–0034). All animals were housed in South China Research Center for Acupuncture and Moxibustion (24°C ± 1°C, 12/12 h light/dark cycle), with sufficient food and water freely available. All animal studies described in this article were approved by the Ethical Committee of Guangzhou University of Chinese Medicine.

On day 25 after birth, the mice were randomly placed into two groups: the control group (*n* = 12) and the DHEA group (*n* = 18). The DHEA group was given DHEA subcutaneously across their back (6 mg/100 g, 100 *μ*l/mouse in sesame oil with 10% of 95% ethanol, [Sigma-Aldrich, St. Louis, MO, USA]) for 20 consecutive days. The control group mice were injected with 90 *μ*l sesame oil and 10 *μ*l 95% ethanol daily for 20 consecutive days. Estrous cycle was monitored daily from day 10 after DHEA treatment until the end of the experiments. After 20 days of treatment, six mice were selected at random from each group to confirm the PCOS model, and the DHEA group was randomly divided into two groups: the DHEA control group (*n* = 6) and the DHEA + EA group (*n* = 6). The DHEA + EA group received EA stimulation at the acupoints Zusanli (ST36), Sanyinjiao (SP6), and Neiguan (PC6). Mice were stimulated with density waves from a Hwato SDZ-III machine at a frequency of 2 Hz and an intensity of 1 mA for 20 min per day for four weeks. When the EA treatment was finished, the mice were fasted overnight, anesthetized, and then sacrificed. Serum samples and left ovaries were collected and stored at −80°C for ELISA and real-time PCR. The left ovaries were dissected for hematoxylin and eosin staining.

### 2.2. Estrous Cycle Detection

Vaginal smear analysis was performed daily at 9 am from 10 days after DHEA treatment. Vaginal cells were collected via saline lavage and dropped onto a glass slide. After air-drying, they were stained with 0.1% methylene blue (Solarbio, China). Estrous stages were determined based on the predominant cell type as described previously [24, 28].

### 2.3. Intraperitoneal Glucose Tolerance Test and Insulin Tolerance Test

After 20 days of DHEA treatment, the mice fasted for 12 h before the experiment. Blood was extracted from the tail vein following disinfection of the tail with 75% ethanol. After measuring the fasting glucose levels at 9 am by ACCU-CHEK Performa (YZB/GER 5513–2014), the mice were given D-glucose (Sigma-Aldrich, 2 g/kg body weight as a 50% glucose stock) through intraperitoneal injection. Blood glucose was then measured at 30, 60, 90, and 120 min.

Three days after the intraperitoneal glucose tolerance test (IPGTT) experiment, mice were fasted for 6 h before intraperitoneal injection with insulin (1 IU/kg body weight, Humalog Mix25, China). Tail blood sampling was performed at 9 am and then at 30, 60, 90, and 120 min after intraperitoneal injection.

### 2.4. Hormone Measurements

At the end of the EA treatment, the mice were anesthetized and blood samples were collected from the orbit. After standing at room temperature for 2 h, blood samples were centrifuged at 2500 rpm for 15 min. The upper serum was extracted and stored at −80°C. Serum estrogen, progesterone, testosterone, follicle-stimulating hormone, luteinizing hormone, and insulin were determined with mice kits (Elabscience, China). The procedures were carried out in accordance with the manufacturer's instructions.

### 2.5. Real-Time PCR

Ovaries were collected and stored immediately at −80°C. Total RNA was extracted from the ovaries using TRIzol reagent (Life Technologies) according to the manufacturer's instructions. cDNA was generated with the PrimeScript reverse transcriptase reagent kit (Perfect Real Time; TaKaRa, Shiga, Japan). Real-time PCR (RT-PCR) was performed using TB Green Premix Ex Taq II (Tli RNase H Plus, TaKaRa). The program used for RT-PCR was as follows: 95°C for 30 s; 40 cycles of 95°C for 5 s and 60°C for 31 s; 95°C for 15 s; 60°C for 1 min; 95°C for 15 s; and 60°C for 15 s. Relative gene expression was calculated using the 2^−ΔΔCT^ method. The primer sequences are AR: forward: TAAAGACATTTTGAACGAGGCC, reverse: GTCAGATATGGTTGAATTGCCC; ER: forward: CTACTACCTGGAGAACGAGC, reverse: GCGTCGATTGTCAGAATTAGAC; PR: forward: TAGTCTCGCCTATACCGATCTC, reverse: CTTCCCTATGAGTGGCTTCTAC; GNRHR: forward: ATTCTCAGCATTGTCTTTGCAG, reverse: GCA-TTGCAGATTAGCATGATGA;GAPDH: forward: GCACCACCAACTGCTTAG, reverse: CAGTGATGGCATGGACTG.

### 2.6. H&E Staining

The left ovaries were fixed with 4% paraformaldehyde at 4°C for 24 h. After tissue dehydration and paraffin embedding, the ovaries were cut and sliced into 5 *μ*m serial sections with a tissue slicer (Thermo Scientific HM340 E, Germany). To get the most considerable sections of each ovary, one out of every five sections was selected. The sections were stained using the hematoxylin and eosin staining kit (Beyotime, China) following the manufacturer's guidelines. Corpora lutea were counted using an optical microscope (Nikon Eclipse E200, Japan).

### 2.7. Statistical Analysis

All statistical analyses were performed in GraphPad Prism (GraphPad Software Inc., San Diego, CA, USA). One-way analysis of variance (ANOVA) followed by Tukey's post hoc test was used to analyze differences among groups. *p* < 0.05 was considered statistically significant.

## 3. Results

### 3.1. Confirmation of the PCOS Model

**Figure 1:**
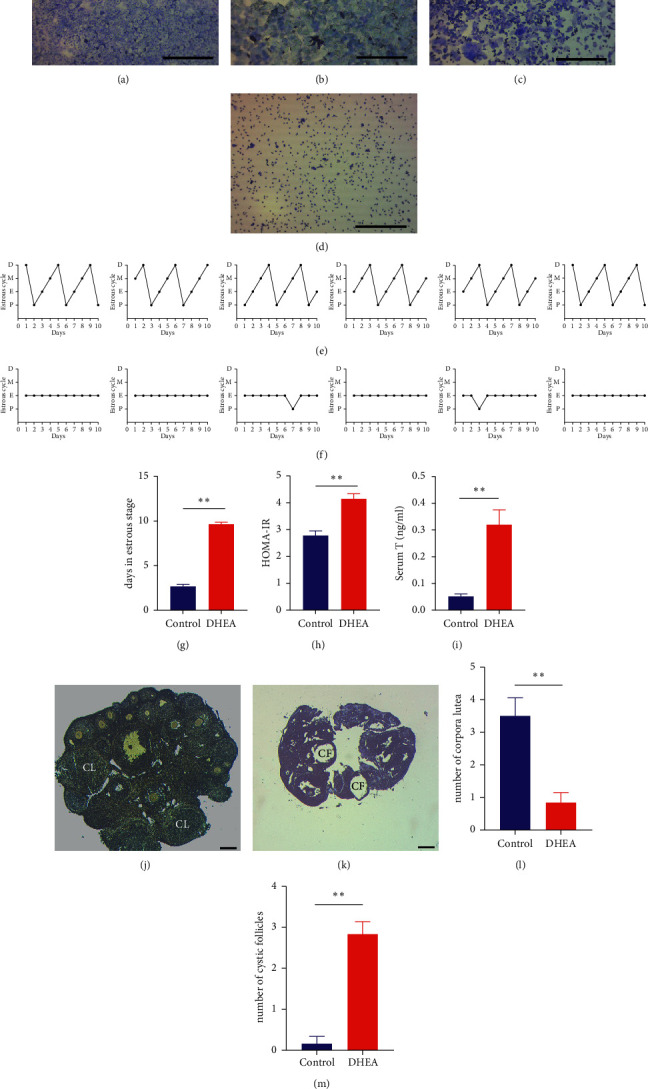
(a) Vaginal smears showing proestrus stage. (b) Vaginal smears showing estrus stage. (c) Vaginal smears showing metestrus stage. (d) Vaginal smears showing diestrus stage. Scale bar = 400 *μ*m. (e) Estrous cycle of the control group. (f) Estrous cycle of the DHEA group. (g) Days in estrus stage. (h) HOMA-IR levels. (i) Serum T levels in mice. (j, k) Representative ovary morphologies from the control and DHEA groups. Scale bar = 200 *μ*m. (l) The number of corpora lutea in ovaries. (m) The number of cystic follicles in ovaries. HOMA-IR: homeostasis model assessment-insulin resistance; T: testosterone; CL: corpus luteum; CF: cystic follicles. ^*∗∗*^*p* < 0.01.

In order to evaluate the establishment of the PCOS model, the estrous cycle was monitored by vaginal smear examination during the experimental period. The control group mice had regular estrus as described previously [24, 28]: a significant number of nuclear epithelial cells appeared to be in the proestrus stage (Figure 1(a)); squamous epithelial cells accounted for the majority of cell types in the estrus stage (Figure 1(b)); nuclear epithelial cells, squamous epithelial cells, and leukocytes were observed in the metestrus stage (Figure 1(c)); and almost all cells were leukocytes in the diestrus stage (Figure 1(d)). The control mice had a regular estrus stage, while in the DHEA group almost all mice were in the estrus stage (Figures 1(e) and 1(f)), which was a typical pathological feature of PCOS. The number of days in the estrus stage increased significantly in the DHEA group (Figure 1(g)). HOMA-IR was calculated (HOMA-IR = FBG (mmol/L) × FINS (mU/L)/22.5) and compared between the control group and the DHEA group after DHEA injection (Figure 1(h)). HOMA-IR was significantly increased in PCOS mice. The DHEA group had higher serum *T* levels than the control mice (Figure 1(i)). Normal ovaries had follicles at various developmental stages and several corpora lutea (CL) (Figure 1(j)), whereas ovaries of the DHEA group had several cystic follicles (CF) (Figure 1(k)).

Compared with the control group, the numbers of CL were significantly decreased (Figure 1(l)) and the numbers of CF were significantly increased (Figure 1(m)). These results indicated that induction of the PCOS model was successful.

### 3.2. EA Improves Estrous Cycle and Serum Hormone Levels

**Figure 2:**
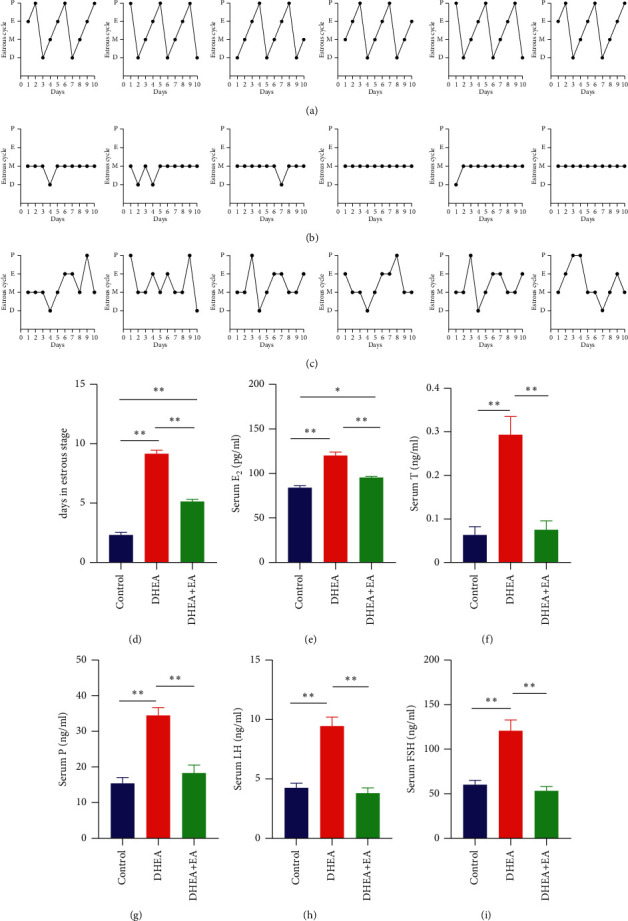
(a) Estrous cycle of the control group. (b) Estrous cycle of the DHEA group. (c) Estrous cycle of DHEA + EA group. (d) Days in estrus stage. (e–i) Serum E_2_, T, P, LH, and FSH levels in different groups of mice. E_2_: estradiol, P: progesterone, T: testosterone, LH: luteinizing hormone, FSH: follicle-stimulating hormone. ^*∗*^*p* < 0.05 and ^*∗∗*^*p* < 0.01.

After EA treatment, we monitored the estrous cycles of the three groups (Figures 2(a)–2(c)). The days of estrus in the DHEA + EA group were significantly reduced compared with the DHEA group but did not recover to the levels of control mice (Figure 2(d)). The average estrogen level in the DHEA group increased by about 1.5 times after treatment with DHEA. EA significantly decreased estrogen levels, although they did not return to control levels (Figure 2(e)). However, DHEA increased progesterone, testosterone, LH, and FSH levels by two to three times, and EA treatment restored them to the control group levels (Figures 2(f)–2(i)). Overall, treatment with EA improved estrous cycle and serum hormone levels.

### 3.3. EA Improves Abnormal Sex Hormone Receptors and Attenuates the Histomorphological Changes of Ovaries Induced by DHEA in Mice

**Figure 3:**
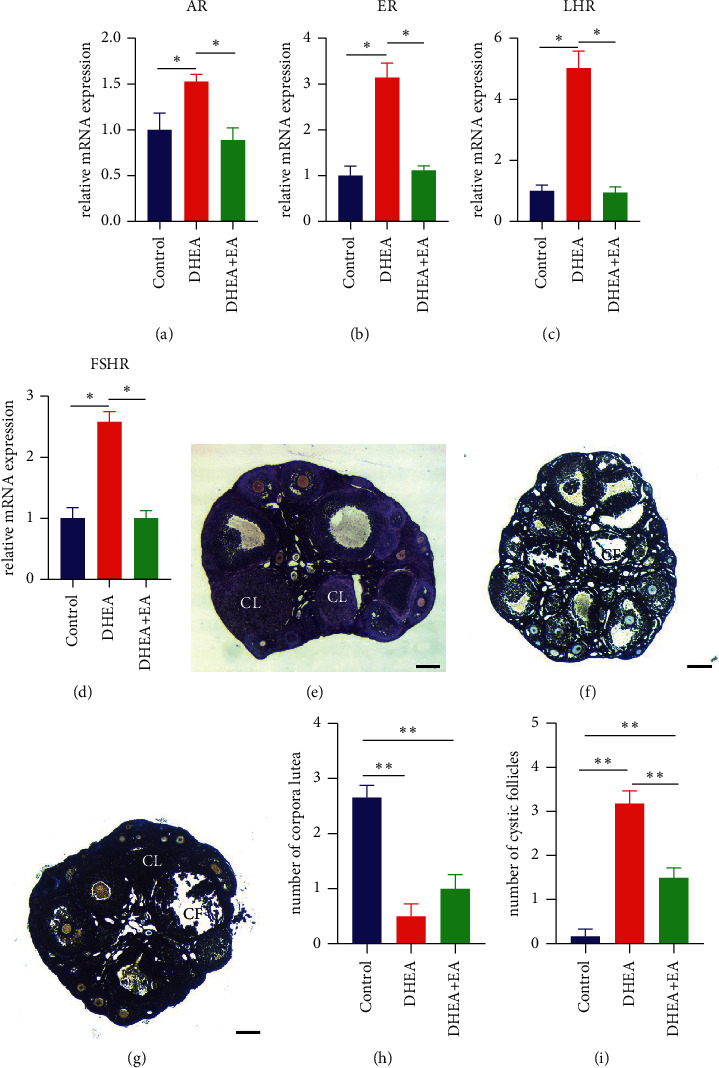
(a–d) AR, ER, LHR, and FSHR gene expressions in the ovaries. (e–g) Representative morphology from control group, DHEA group, and DHEA + EA group. Scale bar = 200 *μ*m. (h) The number of corpus luteum cystic follicles in ovaries. (i) The number of cystic follicles in ovaries. AR: androgen receptor; ER: estrogen receptor; LHR: luteinizing hormone receptor; FSHR: follicle-stimulating hormone receptor. CF: cystic follicles, CL: corpus luteum. ^*∗*^*p* < 0.05 and ^*∗∗*^*p* < 0.01.

The average expression of the AR gene in the DHEA group increased approximately 1.5 times after DHEA treatment. After EA treatment, androgen receptor (AR) gene expression was decreased to the control level (Figure 3(a)). The estrogen receptor (ER) gene in the DHEA group increased approximately three times after DHEA treatment. Following EA intervention, ER expression was comparable to the control group (Figure 3(b)). The average expression of the luteinizing hormone receptor (LHR) gene in the DHEA group increased about six times after DHEA treatment. EA treatment also decreased expression of LHR to a normal level (Figure 3(c)). The average FSHR gene expression was about 2.5 times higher after DHEA treatment and was significantly decreased following EA treatment (Figure 3(d)). Thus, it can be seen that DHEA treatment significantly increased expression of hormone receptor genes in mouse ovaries, which may be an indirect cause of PCOS and which EA treatment can significantly improve. To determine the effects of EA on ovarian morphology, the ovaries of the three groups were stained with hematoxylin and eosin (Figures 3(e)–3(g)). We found that the number of CF was significantly decreased and there was an increasing trend in the number of CL in the DHEA + EA group compared to the DHEA group (Figures 3(h) and 3(i)). The data demonstrate that EA can ameliorate disordered follicular development.

### 3.4. EA Improves Glucose Intolerance, Insulin Intolerance, and HOMA-IR

**Figure 4:**
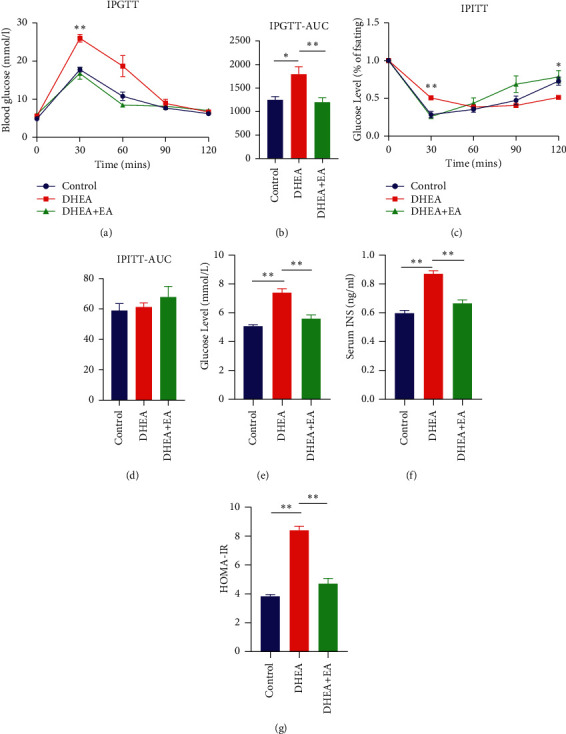
(a) IPGTT experiments. (b) AUC of glucose. (c) IPITT experiments. (d) AUC of glucose. (e) Fasting blood glucose. (f) Serum insulin levels. (g) HOMA-IR levels. IPGTT: intraperitoneal glucose tolerance test; AUC: area under the curve; IPITT: intraperitoneal insulin tolerance test; INS: insulin; HOMA-IR: homeostasis model assessment-insulin resistance. ^*∗*^*p* < 0.05 and ^*∗∗*^*p* < 0.01.

To assess EA efficacy on glucose tolerance and insulin tolerance of the PCOS mice, IPGTT and IPITT were performed. After glucose injection, the blood glucose level of the DHEA group was significantly higher than that of the control group at 30 min (Figure 4(a)). Mice in the DHEA + EA group had a similar blood glucose level at 30 min compared to the control group. Statistical analysis of IPGTT-AUC glucose levels showed a significant increase in the DHEA group mice compared to the control and DHEA + EA groups. There was no significant difference between the control group and the DHEA + EA group (Figure 4(b)). After insulin treatment, blood glucose levels in the DHEA mouse group at 30 min were higher compared to the control group and the DHEA + EA group, and levels did not return to normal by 120 min (Figure 4(c)). However, IPITT-AUC among the three groups was not significantly different (Figure 4(d)). Additionally, fasting blood glucose, serum insulin level, and HOMA-IR were almost doubled in PCOS mice, an effect that was reversed by EA treatment (Figures 4(e)–4(f)).

## 4. Discussion

Because of the complexity of polycystic ovarian syndrome, there is no standardized treatment available; treatment is individualized according to the symptoms and pathogenesis of each patient. For example, acne and hirsutism caused by excess androgen often have psychological repercussions in women with PCOS. Bleaching, plucking, shaving, waxing, chemical treatment, laser hair removal, and electrolysis could improve hirsutism, and dermabrasion, laser therapy, and light therapy can effectively treat acne [29–31]. With impaired glucose tolerance and insulin tolerance, women with PCOS are more prone to obesity, particularly central obesity. With PCOS development, increasing visceral fat deposition could induce high serum androgen production and reduced serum levels of sex-hormone-binding globulin [32]. Compared with normal women, obese women with PCOS are more likely to have metabolic abnormalities and severe cardiovascular conditions [33].

Oxidative stress has a pivotal impact on the biological functions of reproduction and metabolism and also plays a key role in the pathophysiology of PCOS. Excessive mitochondrial production of ROS can damage the function of mitochondria and even cause cell apoptosis. A previous study reported CoQ10 as an antioxidant compound could attenuate ovarian dysfunction induced by cyclophosphamide. CoQ10 reduced ROS levels and increased expression of the proliferation cell nuclear antigen (PCNA) and FSHR genes [34]. Another study reported that treatment with *Panax ginseng* extract (100 *μ*g/mL) as an antioxidant not only had a positive effect on growth of isolated preantral follicles from the ovaries of prepubertal mice but also increased the expression of PCNA and FSHR genes [35]. Fariba Khodaeifar et al. revealed that treatment with *Apium graveolens* and *Cinnamon zeylanicum* could mitigate ovarian tissue injury in rats induced by PCOS. Serum levels of FBS, insulin, triglyceride, low-density lipoprotein, and cholesterol all showed obvious improvement [36].

Although the pathological mechanism of PCOS has not been clarified, most evidence has indicated that it has a close relationship with obesity and insulin resistance [37]. Considering the relations among IR, obesity, and cardiovascular disease, treating IR is a priority, especially in obese women with PCOS. Lifestyle modifications such as reasonable diet and increased exercise should be recommended to women with PCOS, especially those who also have IR or obesity. It is noteworthy that some studies have reported that oral antiobesity drugs resulted in initial positive changes in the signs and symptoms of PCOS; however, their long-term effectiveness remains unknown [38–40]. Bariatric surgery is a desirable treatment for women with IR and PCOS. A meta-analysis reported that this surgery could decrease weight and hirsutism in PCOS patients [41]. However, these therapies could not effectively improve IR symptoms, and they lack validation from long-term studies. Additional treatments for PCOS should therefore still be tested.

Acupuncture plays an essential role in traditional Chinese medicine, where it is widely accepted and used to treat many diseases, mainly because of the absence of side effects compared to many medicines. Electroacupuncture (EA) is a new treatment comprising traditional acupuncture combined with pulsed electrical stimulation. In recent years, the mechanism underlying the effect of EA has been widely studied. A randomized controlled trial found that, after 16 weeks of low-frequency EA treatment, body weight, body mass index, waist-hip ratio, oral glucose tolerance test, insulin release, and glucose and lipid metabolism indicators such as total cholesterol, triglycerides, and high-density lipoprotein cholesterol decreased compared with the baseline. The results show that EA improves insulin resistance in PCOS patients by regulating glucose and lipid metabolism [42]. The three acupoints used in this study are a common combination often used to treat metabolic diseases. After consulting the literature, we found that SP6 and ST36 are also very effective in the treatment of PCOS [22, 43, 44]. Furthermore, PC6 has the ability to clear meridians and regulate qi and xue in Chinese medicine, which is why we choose these three acupoints.

In this study, we successfully established a mouse model of PCOS with irregular estrous cyclicity; excessive estrogen, progesterone, and testosterone in serum; and increased plasma insulin, LH, and FSH levels. The pathological changes of ovaries were as follows: the number of corpora lutea and mature oocytes decreased, atresia follicles appeared, and expressions of AR, ER, PR, and GNRHR were increased. After EA treatment, excessive serum hormones and sex hormone receptors returned to normal levels, and ovarian morphology tended to improve. We also conducted IPGTT and IPITT experiments, and the results suggested that, after EA treatment, mice with PCOS were more sensitive to glucose and insulin. Thus, EA had a positive effect on IR caused by PCOS.

The hypothalamus-pituitary-ovary axis regulates reproductive functions of female mice. In PCOS, the massive LH stimulated ovarian theca cells lead to production of more androgen. In ovaries, follicles are not sensitive to FSH, which may be a critical factor in PCOS. This further causes reduced conversion efficiency of androgens to estrogens, leading to hyperandrogenism.

However, the relationship between IR and PCOS is unknown. Some studies have reported the mechanism of EA in the treatment of IR in PCOS. For instance, EA treatment could activate the activated protein kinase (AMPK) pathway in PCOS rats, inhibiting high expression of sterol regulatory element-binding protein-1 (SREBP1). Phosphorylation of insulin-stimulated insulin receptor *ß* and serine/threonine-specific protein kinase (AKT) returned to normal in primary granulosa cells [22].

Proopiomelanocortin (POMC) neurons in the arcuate nucleus of the hypothalamus (ARC) not only are a central regulator of energy homeostasis but also play a role in reproductive circuits. POMC neurons in mice with IR develop perturbations in maintenance of glucose metabolism and leptin sensitivity [45]. It is possible that EA improves IR by regulating POMC neurons. Research has found that GnRH neurons in the preoptic area express receptors for *ß*-endorphin [46]. Furthermore, some POMC neurons in the ARC express Er*α* [47], and these neurons have been demonstrated to project to the preoptic area (POA), where GnRH neurons are located [48]. *ß*-Endorphin in rats could inhibit GnRH and LH secretion [49]. *α*-Melanocyte-stimulating hormone has an excitatory effect on the GnRH neuron by inducing central melanocortin receptors [50]. POMC neurons also regulate GnRH neurons by releasing *γ*-aminobutyric acid (GABA) and glutamate [51]. GABA is the main inhibitory neurotransmitter of the central nervous system. A previous study showed that mice with PCOS increased GABA innervations to GnRH neurons in the hypothalamus [52]. *GABAB1* knockdown mice had a disordered estrus cycle and decrease in fecundity pulse frequency [53]. A meta-analysis suggested that valproate, a GABA-agonist drug, can be used to treat epilepsy, which has close associations with the development of PCOS. These results show that GABA neurons likely play an important role in gonadotropin hormones changes in PCOS [54]. It has also been suggested that EA could decrease *ß*-endorphin concentration in both the hypothalamus and peripheral blood and increase ovarian blood and ovarian nerve growth factor (NGF) [43, 55, 56]. It is likely that EA recovers GNRH pulse by regulating *ß*-endorphin and improving the concentration of hormones secreted by the ovaries.

In the present study, we demonstrated that EA has a considerable effect on DHEA-induced PCOS mice. However, our study did not address the mechanism of acupuncture in treating PCOS. We did not examine the effect of acupuncture on neuronal sensitivity to glucose and did not examine changes in insulin receptor and leptin receptor on POMC neurons. In future studies, we will focus on how EA treatment improves IR through POMC neurons.

## 5. Conclusions

In summary, our study suggests that EA has an ameliorative effect on PCOS model mice induced by DHEA. EA can improve estrous cycle, hormonal disorders, abnormal ovarian sex hormone receptors, ovarian morphology, and insulin resistance in PCOS mice. Therefore, EA may represent a valid therapeutic option for women with PCOS.

## Data Availability

Data used for analyses in this study are available from the corresponding author upon reasonable request.

## Abbreviations

EA: Electroacupuncture
DHEA: Dehydroepiandrosterone
AUC: Area under the curve
IPGTT: Intraperitoneal glucose tolerance test
IPITT: Intraperitoneal insulin tolerance test
LH: Luteinizing hormone
FSH: Follicle-stimulating hormone
HOMA: Homeostasis model assessment
IR: Insulin resistance
FSHR: Follicle-stimulating hormone receptor
LHR: Luteinizing hormone receptor
AR: Androgen receptor
ER: Estrogen receptor
GnRH: Gonadotropin-releasing hormone
AMPK: AMP-activated protein kinase
SREBP1: Sterol regulatory element-binding protein-1
AKT: Serine/threonine-specific protein kinase
POMC: Proopiomelanocortin
ARC: Arcuate nucleus of the hypothalamus
PCNA: Proliferation cell nuclear antigen
ROS: Reactive oxygen species.
